# Prevalence and associated factors of maternal depression among mothers of children with undernutrition at comprehensive specialized hospitals in Northwest Ethiopia in 2023: a cross−sectional study

**DOI:** 10.3389/fpsyt.2024.1400293

**Published:** 2024-07-03

**Authors:** Biazin Yenealem, Misrak Negash, Derebe Madoro, Alemayehu Molla, Goshu Nenko, Girum Nakie, Berhanie Getnet

**Affiliations:** ^1^ Department of Psychiatry, College of Health and Medical Science, Dilla University, Dilla, Ethiopia; ^2^ Department of Psychiatry, College of Health and Medical Science, Injibara University, Injibara, Ethiopia; ^3^ School of Health, Faculty of Medicine and Health Science, University of New England, Armidale, NSW, Australia; ^4^ Department of Psychiatry, College of Medicine and Health Science, University of Gondar, Gondar, Ethiopia

**Keywords:** prevalence, maternal depression, malnutrition, undernourished children, Ethiopia

## Abstract

**Background:**

Malnutrition is one of the most significant child health problems in developing countries, accounting for an estimated 53% of child deaths per year. Depression is the leading cause of disease-related disability in women and adversely affects the health and well-being of mothers and their children. Studies have shown that maternal depression has an impact on infant growth and nutritional status. However, evidence is scarce regarding the relationship between maternal depression and child malnutrition.

**Objectives:**

The general objective of this study was to assess the prevalence and associated factors of maternal depression among mothers of undernourished children at comprehensive specialized hospitals in Northwest Ethiopia in 2023.

**Methods:**

An institution-based cross-sectional study was conducted among 465 participants. Outcome variables were assessed using a Patient Health Questionnaire-9 (PHQ-9). Data were analyzed using SPSS-25. Bivariate and multivariable logistic regression analyses were conducted. Variables with a p-value less than 0.05 were considered statistically significant with a corresponding 95% confidence interval (CI).

**Results:**

The prevalence of maternal depression among mothers of children with undernutrition was 36.4% (95% CI = 32%–41%). According to a multivariate analysis, lack of maternal education (adjusted odds ratio [AOR] = 2.872, 95% CI = 1.502–5.492), unemployment (AOR = 2.581, 95% CI = 1.497–4.451), poor social support (AOR = 2.209, 95% CI = 1.314–3.713), perceived stigma (AOR = 2.243, 95% CI = 1.414–3.560), and stunting (AOR = 1.913, 95% CI = 1.129–3.241) were factors significantly associated with maternal depression.

**Conclusion:**

The overall prevalence of maternal depression was higher among mothers of children with undernutrition. This higher prevalence was associated with several factors, including lack of education, unemployment, poor social support, high perceived stigma, and stunted physical growth in the children themselves. To decrease maternal depression, we can address these factors by increasing the level of maternal education and employment opportunities, strengthening social support systems, reducing stigma, and providing interventions to reduce stunting.

## Introduction

Maternal depression refers to depressive disorders experienced by pregnant women and mothers up to 12 months after giving birth. Prenatal, postpartum, and psychotic disorders are all possible manifestations of maternal depressive illness (1). Symptoms of maternal depression are emotional and psychological changes that can affect mothers during pregnancy and after childbirth. These symptoms include distress, a depressed mood, feeling sad, and loss of interest or pleasure, and these can interfere with a mother’s responsiveness and ability to care for herself and her baby (2–4).

Malnutrition is defined as a condition where a person’s intake of energy and/or nutrients is deficient, excessive, or unbalanced. Undernutrition is a category of malnutrition that includes stunting (low height for age), wasting (low weight for height), and being underweight (low weight for age) (5). Since feeding and caring for young people are primarily the responsibility of mothers, poor maternal physical or mental health can adversely affect the nutritional, health, and psychological well-being of children (6).

Malnutrition is one of the most significant child health issues in developing countries, with approximately 19.4% and 29.9% of children aged less than 5 years being underweight and stunted, respectively (7). The rate of malnutrition among children in Ethiopia ranges from 27% to 37% (8). In a study of malnourished children, stunting (57.1%) was the most common form of malnutrition, followed by undernutrition (49.2%) and wasting (42.3%) (9). Maternal depression affects 15.6% and 19.8% of women during the antenatal and postnatal periods, respectively, in low- and middle-income countries (LMICs) (2). In sub-Saharan African countries, the rate of maternal depression ranges from 6% to 30%. Around 20% of women experience depression symptoms during pregnancy, and over 40% of the symptoms persist after delivery. Additionally, 80% of women with postpartum depression are likely to experience future depressive episodes (10). In Ethiopia, the prevalence of maternal depression ranges from 12.2% to 33.8%, and the overall prevalence of postpartum depression among mothers was 22.08% (11). Maternal depression is prevalent, affecting an estimated 14% of new mothers in the first 6 months after delivery (12). Studies from different countries have shown that the prevalence of depression was higher among mothers of children with undernutrition than among mothers of children with a normal nutritional status; the reported evidence from Uganda indicates that the prevalence of depression among mothers of malnourished children was 42%, while it was 13% among mothers in the control group (13). In Kenya, 64.1% of mothers with malnourished children experience depression, which was higher than 5.1% of mothers of children with normal weight (6). In Ethiopia, the prevalence of maternal depression among mothers of malnourished children was 22.8% (14).

Although the prevalence of child malnutrition in many regions of the world has declined in recent years, it remains one of the most significant child health problems, affecting an estimated 53% of child deaths per year (15, 16).

Malnutrition impairs physical growth, increases morbidity and mortality, and reduces both cognitive development and physical work capacity (17). Malnutrition is an underlying risk factor that increases the morbidity and mortality of many diseases in children and adults (18). Research in developing countries indicates that maternal depression is a risk factor for poor child growth in young infants (19).

Additionally, the interplay between women’s susceptibility to depression, their primary childcare responsibilities, and the high rates of maternal depression in developing countries significantly impact child development (20). Moreover, psychosocial factors, including maternal mental health status, affect breastfeeding in both high- and low-income countries (21–23). Mothers with depression symptoms also stop exclusive breastfeeding earlier than mothers without symptoms (22, 24).

Maternal mental health significantly impacts children’s nutritional status by interfering with a mother’s ability to fulfill her childcare responsibilities (25, 26). Evidence suggests a link between maternal mental health and child undernutrition in low-income countries, with an increased risk of stunting of more than 40% for children of mothers with depression symptoms (13, 27, 28). Risk factors for maternal depression include low income, education level, lack of financial support, child age, residence, maternal education, household wealth index, sex, and family size (29–32).

Even though the impact of maternal mental health on children’s emotional, cognitive, and behavioral problems has been well-studied in high-income countries, it has received less attention in LMICs, where malnutrition presents a significant additional challenge (33, 34).

Despite limited research on how child factors influence maternal depression, there is also a scarcity of studies examining the link between maternal depression and child health problems like malnutrition and illness. To address this gap, this study aimed to assess the prevalence and associated factors of maternal depression among mothers of malnourished children admitted to comprehensive specialized hospitals in Northwest Ethiopia.

## Objective

### General objective

The general objective of this study was to assess the prevalence and associated factors of maternal depression among mothers of undernourished children at comprehensive specialized hospitals in Northwest Ethiopia in 2023.

### Specific objectives

The study’s specific objectives were the following: to determine the prevalence of maternal depression among mothers of undernourished children at andto identify the factors associated with maternal depression among mothers of undernourished children at comprehensive specialized hospitals in Northwest Ethiopia in 2023.

## Methodology

### Study design and study area

A cross-sectional study was conducted from March to April 2023 at three comprehensive specialized hospitals in Northwest Ethiopia. The University of Gondar Comprehensive Specialized Hospital (UoG CSH) is located in Gondar, 750 km northwest of Addis Ababa, the capital city of Ethiopia. The other two hospitals, Tibebe Ghion Comprehensive Specialized Hospital (TGCSH) and Felege Hiwot Comprehensive Specialized Hospitals (FHCSH), are situated in Bahir Dar City, 565 km from Addis Ababa. Bahir Dar is the capital of the Amhara Regional State. All three hospitals have dedicated rooms for treating malnourished children, with an estimated 1,042 children receiving treatment each month across the pediatric wards and outpatient departments (OPDs).

### Source population

All mothers of children with undernutrition who are receiving nutritional supplements and treatment at comprehensive specialized hospitals in Northwest Ethiopia.

### Study population

All mothers of children with undernutrition who are receiving nutritional supplements and treatment at comprehensive specialized hospitals in Northwest Ethiopia and who availed at the time of the study period.

### Eligibility criteria

#### 3.4.1 Inclusion and exclusion criteria

During the data collection period, mothers of malnourished children attending the pediatric wards and OPDs were included in the study. However, participants who were critically ill, had difficulty communicating, and had pre-existing depression were excluded.

### Sample size determination

The sample size was determined by using a single population proportion formula with a 95% confidence interval (95% CI) level and a 4% margin of error and considering the prevalence of maternal depression [22.8%, proportion (P) = 0.228] from a study conducted in Ethiopia (14).


$$\frac{{\left(\frac{Z\alpha }{2}\right)}^{2\;}p\left(1-P\right)}{d^{2}}$$

where n = the minimum sample size
Z critical value = 1.96
P = 22.8%
95% CI and 4% margin of error

$$\frac{\text{n}={\left(1.96\right)}^{2}\times 0.228\times 0.772}{{\left(0.04\right)}^{2}}=423$$

After adding a 10% nonresponse rate (42), the final sample size was 423 + 42 = 465 participants.

### Sampling technique and procedure

Northwest Ethiopia has three comprehensive specialized hospitals, namely, University of Gondar Comprehensive Specialized Hospital (UOG CSH, Tibebe Ghion Comprehensive Specialized Hospital (TGCSH, and Felege Hiwot Comprehensive Specialized Hospital (FH CSH). On average, an estimated 1,042 mothers of undernourished children visited the three hospitals (UOG CSH: 498, FH CSH: 170, TG CSH: 374) per month during the data collection period.

**Figure 1:**
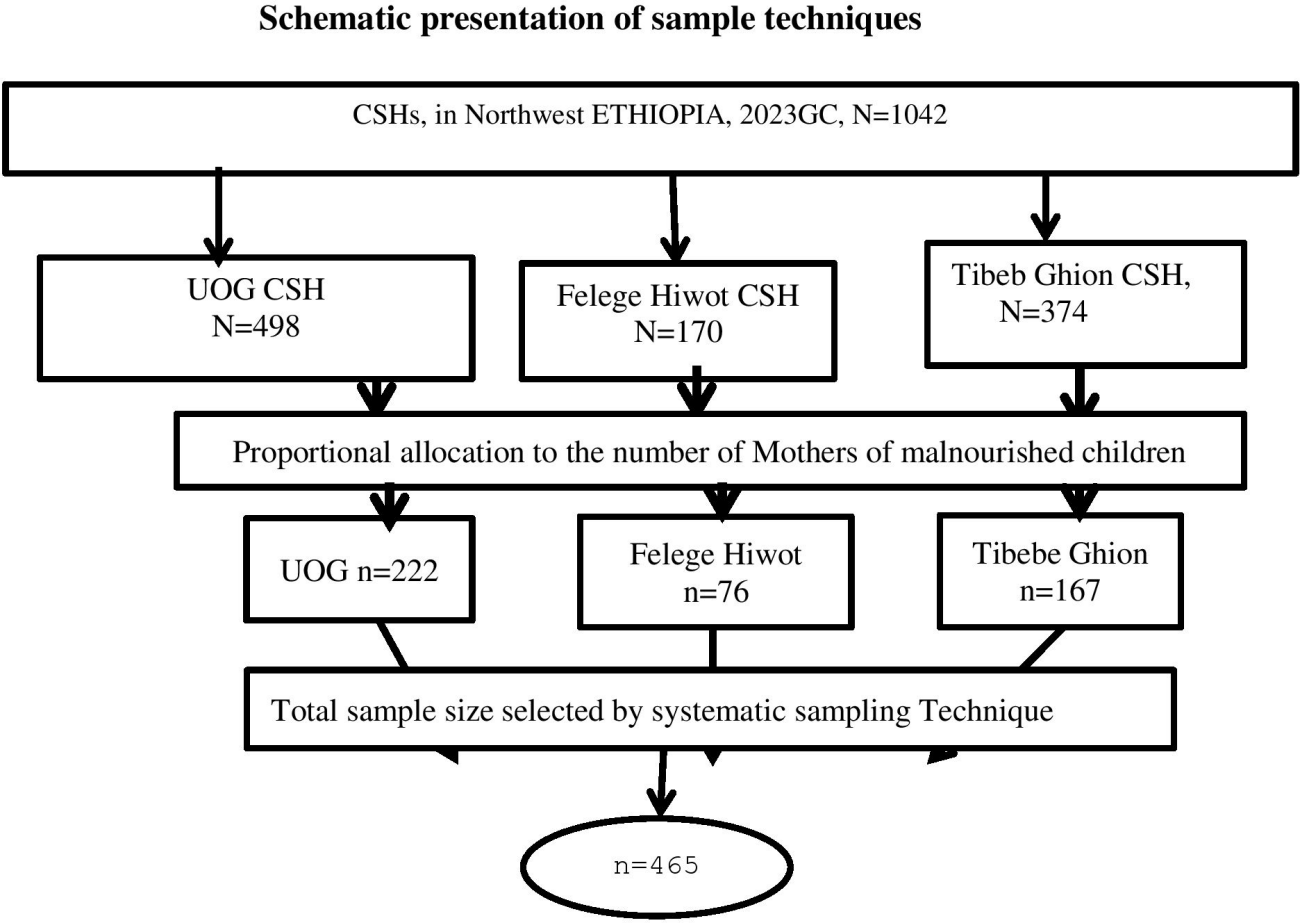
Showing sampling technique and procedure for Prevalence of maternal depression and associated factors among children with under nutrition in comprehensive specialized hospital in Northwest, Ethiopia 2023.

A systematic random sampling technique was used with a sampling interval of 2. This interval was calculated by dividing the total study population (n = 1,042) to the sample size (N = 465). After the proportional allocation of patients to the three hospitals, samples were selected at random points within the designated intervals (**Figure 1**).

### Variables

#### Dependent variable

Maternal depression: Yes/No.

#### Independent variables

Socio-demographic factors: Age, residence, marital status, education level, occupation, and family size were assessed by a structured questionnaire.
Clinical factors for mothers: History of chronic medical illness and family history of mental illness were considered.
Substance use factors: Current substance use (alcohol or khat) was considered.
Child-related factors: Age, sex, medical illness status, weight, and height were included.
Obstetric factors: Pregnancy complications or illnesses, unplanned pregnancy, mode of delivery, stressful life events during pregnancy, undesired child sex, and history of abortion were included.
Psychosocial factors: Social support (husband support), domestic violence (intimate partner violence), unsatisfactory relationships with mothers-in-law, unsatisfactory relationships with husbands, and perceived stigma were included.
Child-related factors: A child’s age, history of medical illness, and nutritional status (height and weight) were included.

### Data collection instrument and procedure

The data were collected by reviewing patient charts and carrying out an interviewer-administered questionnaire, which was adapted to local contexts employed as a tool for this study.

Socio-demographic characteristics: Residence, age, marital status, ethnicity, religion, education, and occupational status were included.
Maternal depression: Patient Health Questionnaire-9 (PHQ-9) was used to measure maternal depression. The PHQ-9 is a screening tool created in 2001 by Dr. Robert Land and colleagues at Columbia University in the US to screen adult patients for the presence and severity of depression in a primary care setting (35). The PHQ-9 is a self-rated depression scale with nine items that ask about the past 2 weeks, with response options ranging from “not at all” to “nearly every day.” These items correspond to the nine criteria used to diagnose major depressive disorder in the DSM-5 (35). The PHQ-9 score ranges from 0 to 27. The scores of the nine items range from 0 (not at all) to 3 (nearly every day) (35). The PHQ-9 is used to grade depressive symptom severity as none (score of 0–4), mild (score of 5–9), moderate (score of 10–14), moderately severe depression (score of 15–19), and severe depression (score of 20–27) (35). The PHQ-9 has validity and usefulness in East Africa; it is widely used in Kenya for screening maternal depression (36) and in rural settings in Ghana for screening postpartum depression (37). The PHQ-9 items showed good internal consistency (Cronbach’s alpha = 0.85) (38). The PHQ-9 has been validated in Ethiopia, both in urban and rural areas. The cut-off points differ, with a score of ≥10 indicating depression in urban settings and a score of ≥5 in rural settings. In urban areas, the PHQ-9 shows a specificity of 67% and a sensitivity of 86%. In rural settings, the sensitivity is 83.5%, and the specificity is 74.7% (38, 39).
Social support: This was measured using the Oslo Social Support Scale (OSSS-3) (40). It is a quick and affordable tool for determining the extent of social support and consists of three items, the sum of which ranges from 3 to 14 (41). The OSSS-3 scores poor social support (3–8 points), intermediate social support (9–11 points), and strong social support (12–14 points). In African countries, it has been validated in Nigeria among the population of clinical students and in general adult depression patients. The Oslo-3 score is commonly used to assess the level of social support in different Ethiopian studies, particularly related to psychiatric conditions. In Nigeria, the internal consistency (Cronbach’s alpha) of the Oslo-3 tool has a value of 0.91 (42).
Perceived stigma: This was assessed by a three-item stigma scale. In the European population, a simple three-question tool for determining the extent of patients’ perceived stigma has been developed and validated (43). In African countries, a three-item stigma scale was validated in Zambia specifically for epilepsy patients. This scale uses yes-or-no questions to assess their perceived stigma. A score of 0 indicates no perceived stigma, while 1 to 3 indicates perceived stigma (43).
Intimate partner violence: This is defined as violence inflicted by current or former spouses, boyfriends, girlfriends, or ex-partners who can be of any legal marital status. Women were considered to have experienced intimate partner violence (IPV) if they answered “yes” to any of the following: a single instance or a combination of sexual, psychological, or physical coercive acts (44).
Clinical factors of the mothers: Family history of mental illness and history of medical illness were assessed by structured questionnaires.
Child-related factors: Age, sex, childhood medical illness, and undernutrition status were the factors assessed here.

#### Anthropometric measurements

Children’s ages were recorded, and anthropometric measurements [mid-upper arm circumference (MUAC), weight, and height] were taken following standard procedures (45).

This study assessed undernutrition through the following indicators: stunting, being underweight, and wasting. For newly admitted children, weight, height, and mid-upper arm circumference (MUAC) were measured using standard anthropometric techniques. For long-term hospitalized children receiving established nutritional support, anthropometric data were retrieved from medical charts. All anthropometric data were converted into Z-scores for height-for-age, weight-for-height, and weight-for-age. Children with Z-scores below -3 standard deviations (SD) and a MUAC of less than 115 mm, according to WHO growth standards, were classified as undernourished (46).

Children with weight to age, height to age, and weight to height Z-scores (WAZ, HAZ, and WHZ, respectively) greater than or equal to -2 standard deviations (≥ -2 SD) indicate normal underweight, stunted, and wasted, respectively (47, 48).

Children with WAZ, HAZ, and WHZ between -2 SD and -3 SD indicate mild underweight, stunted, and wasted, respectively (47, 48).

Children with WAZ, HAZ, and WHZ less than -3 SD indicate moderately/severely underweight, stunted, and wasted, respectively (47, 48).

Obstetric factors: Factors such as unplanned pregnancy, mode of delivery, history of abortion, birth complications, child hospitalization, desired sex of the child, and child death were assessed by structured questionnaires.
Substance-related factors: These included current use and every use of substance.
Current substance use: Alcohol, Smoking, and Substance Involvement Screening Test (ASSIST) indicates the use of at least one specific substance (alcohol or khat) for nonmedical purposes within the past 3 months (49).
Ever use of substances: Alcohol, Smoking, and Substance Involvement Screening Test (ASSIST) indicates that at least one specific substance, such as alcohol or khat, is used for nonmedical purposes at least once in a lifetime (49).

### Operational definitions

Maternal depression: Patients who were found to score >10 on the PHQ-9 were considered to have depression (35).
Social support: This was assessed using the Oslo scale and was categorized as poor (a score of 3–8 points), moderate (a score of 9–11 points), or strong (a score of 12–14 points).
Perceived stigma: Mothers’ perceived stigma is categorized based on their scores on a three-item stigma scale measured on a four-point Likert scale (0, 1, 2, and 3). A score of 0 indicates no perceived stigma, while a score of 1 or higher indicates perceived stigma (43).
Intimate partner violence: Among current or past intimate partners, women were considered to have experienced intimate partner violence (IPV) if they answered “yes” to any one or a combination of the following ranges of sexual, psychological, or physical coercive acts (44).

#### Child anthropometry

Children were classified as undernourished if the Z-scores of all three anthropometric indicators (weight-for-age, height-for-age, and weight-for-height) fell below -3 standard deviations (SDs) of the median WHO growth criteria. Additionally, children with WAZ, HAZ, and WHZ less than -3 were classified as underweight, stunted, and wasted, respectively (47, 48).

Ever use of substance: This refers to the use of at least one of any specific substances for a nonmedical purpose at least once in a lifetime (alcohol or khat).
Current substance use: This was defined as the use of at least one specific substance for nonmedical purposes within the last 3 months (alcohol or khat).
Chronic medical illness: This refers to previous experiences of any physical illness whose information was collected using the following question: “Have you ever been faced with any known chronic medical illness?” If their response was yes, then it was considered to indicate a chronic medical illness.
Family history of mental illness: A history of maternal mental health problems was determined on the basis of this guiding question: “Do you have family members who have ever known about mental illness?” If their response was “yes”, then the respondent was considered to have a family history of mental illness.

### Data collection process and data quality assurance

Data collection involved both face-to-face interviews and reviews of children’s medical charts. An Amharic version of the pretest was administered to a subsample of approximately 5% (n = 23) of the total sample at the Debre Tabor University referral hospital. The Cronbach’s alpha for this subsample (PHQ-9) was 0.76.

Prior to data collection, data collectors and supervisors received 2 days of training on the study’s objectives, sampling procedures, the use of structured questionnaires, and ethical considerations including confidentiality. Regular supervision and monitoring were provided by the supervisor and principal investigator to ensure data quality. The collected data underwent a thorough review for completeness and consistency. Mothers identified as highly depressed during the study were advised to seek mental health services at nearby hospitals.

### Data processing and analysis

First, the data were checked for completeness and consistency. The collected data were coded, edited, entered, and checked into the computer using EPI data version 4.6 and analyzed using the SPSS version 25. The results were presented in numbers, frequencies, tables, charts, and figures.

To assess the association between the dependent and independent variables, an adjusted odds ratio was calculated using logistic regression. The significance level was determined at a 95% confidence interval. Additionally, both bivariate and multivariate logistic regression analyses were conducted to identify independent predictors of maternal depression. This involved entering each independent variable separately into a bivariate analysis. Subsequently, variables with a p-value of less than 0.2 in the bivariate analysis were entered into the multivariate logistic regression model. Variables demonstrating a statistically significant association with a p-value less than 0.05 were considered to be predictors of maternal depression.

Finally, the model’s fitness was evaluated, revealing a Hosmer and Lemeshow test value of 0.65. Tolerance and variance inflation factors (VIFs) were also examined to assess multicollinearity. This analysis confirmed that the tolerance was ≥0.253, while the VIF was ≤4.924.

## Results

### Socio-demographic characteristics of the study participants

A total of 465 mothers of children with undernutrition participated in this study. Of these participants, 456 (98%) completed the interview. The remaining nine participants were excluded due to incomplete data. The participants’ ages ranged from 19 to 42 years. The mean age of the respondents was 30.22 years (SD: ± 5.292). Nearly two-thirds (65.4%) of the study participants were aged between 25 and 34 years. Over three-fourths of the respondents were married 361 (79.2%). Approximately 83 (18.2%) of the participants had not attended formal education. Nearly 177 (40%) of the mothers were housewives, and the majority 256 (56.1%) were urban residents (**Table 1**).

**Table 1:** Socio-demographic characteristics of participants among mothers of children with undernutrition in Northwest Ethiopia in 2023 (n = 456).

Variables	Categories	Frequency	Percent
Age in years	16–24	35	7.7
	25–34	298	65.4
	>34	123	27
Mothers’ marital status	Married	361	79.2
	Divorced	85	18.7
	Widowed	10	2.2
Mothers’ educational status	Not educated	83	18.2
	Primary	183	40.1
	Secondary and above	190	41.7
Mothers’ occupation	Employed	151	33.1
	Housewife	177	38.8
	Unemployed	128	28.1
Residence	Urban	256	56.1
	Rural	200	43.9

Housewife: In this study, a housewife is a woman who manages the home and family full-time, usually without an external paying job and depends financially on her spouse.
Unemployed: This denotes without a job but actively seeking work; in this study, these are qualified women (including college graduates) ready for future opportunities.

### Psychosocial and clinical characteristics of the participants

The majority [192 (42.1%)] of the respondents reported having poor social support. Nearly half (48.9%) of the participants experienced perceived stigma due to their children’s undernutrition. Nearly half (44.7%) of mothers reported experiencing intimate partner violence. Nearly one-fifths (18.4%) of mothers experienced both physical and verbal abuse. Over two-fifths (43.2%) of mothers expressed dissatisfaction with their marital relationships.

Mental and physical health concerns were also present among the participants. About 46 (10.1%) of the mothers had a family history of mental illness, and 35 (7.7%) of the mothers had a known chronic medical illness (**Table 2**).

**Table 2:** Psychosocial and clinical characteristics of the participants among mothers of children with undernutrition in Northwest Ethiopia in 2023 (n = 456).

Variables	Categories	Frequency	Percent
Social support	Poor social support	192	42.1
	Moderate social support	144	31.6
	Good social support	120	26.3
Perceived stigma	Yes	223	48.9
	No	233	51.1
Intimate partner violence	Yes	204	44.7
	No	252	55.3
	Verbal and physical	84	18.4
	Physical	32	7.0
	Verbal	70	15.4
	No abuse	270	59.2
Satisfaction with marital relationship	Yes	259	56.8
	No	197	43.2
Family mental illness	Yes	46	10.1
	No	410	89.9
Chronic medical illness	Yes	35	7.7
	No	421	92.3
	DM	26	5.7
	HIV AIDS	9	2.0

### Substance-related characteristics of participants

Nearly one-third (32.5%) of the study participants used substances at least once in their lifetime, whereas nearly one-third (31.4%) of the mothers reported drinking alcohol at least once in their lifetime. Among the study participants, one-fourth (25.9%) of the mothers used substances in the past 3 months. Eighty-four (18.4%) of the mothers drank alcohol in the current and past 3 months (**Table 3**).

**Table 3:** Substance-related characteristics of the participants among mothers of children undernutrition in Northwest Ethiopia in 2023 (n = 456).

Variables	Categories	Frequency	Percentage
**Every substance used in life**	Yes	148	32.5
	No	308	67.5
Alcohol	Yes	143	31.4
	No	313	68.6
Khat	Yes	17	3.7
	No	439	96.3
Alcohol and khat	Yes	7	1.5
	No	449	98.5
**Current substance used**	Yes	118	25.9
	No	331	72.6
Alcohol	Yes	84	18.4
	No	372	81.6
Khat	Yes	29	6.4
	No	427	93.6
Alcohol and khat	Yes	11	2.5
	No	445	97.5

### Obstetric factors

Four-fifths (81.1%) of the respondents reported having two to four pregnancies. Nearly one-fifth (17.1%) of the children had a prior hospitalization. A total of 58 (12.7%) mothers had a history of abortion. A total of 42 women (9.2%) delivered via caesarean section (C section). Nearly three-fourths (77.4%) of pregnancies were unintended. A total of 44 (9.6%) mothers reported delivery complications during their last pregnancy (**Table 4**).

**Table 4:** Obstetric factors of the participants among mothers of children with undernutrition in Northwest Ethiopia in 2023 (n = 456).

Variable	Categories	Frequency	Percentage
Number of mother’s pregnancies	1	67	14.9
	2–4	370	81.1
	>4	18	4
Number of abortions	Yes	58	12.7
	No	398	87.3
Hospitalization of baby	Yes	78	17.1
	No	378	82.9
Death of children	Yes	46	10.1
	No	410	89.9
Mode of delivery	Vaginally	357	78.3
	Operation	42	9.2
	Instrumental or C-section	57	12.5
Planned pregnancy	Yes	103	22.6
	No	353	77.4
Sex of the last child	Male	297	65.1
	Female	159	34.9
Desired sex for the last child	Yes	385	84.4
	No	71	15.6
Complications during last pregnancy	Yes	44	9.6
	No	412	90.4

### Anthropometric measurements of children

Among children with undernutrition, two-thirds (75.7%) were underweight (low weight for age), 62.1% were stunted (low height for age), and 50.7% were wasted (low weight for height). Additionally, 5.7% of the children with malnutrition had known chronic medical diseases, such as type 1 diabetes mellitus (DM1), human immunodeficiency virus (HIV), or congestive heart failure (**Table 5**).

**Table 5:** Anthropometric measurements and clinical characteristics of children among mothers of children with undernutrition in Northwest Ethiopia in 2023 (n = 456).

Variables	Categories	Frequency	Valid Percent
Age of child in months	0–5	56	12.3
	6–23	185	40.6
	24–59	215	47.1
WAZ (weight to age) (underweight)	Yes	345	75.7
	No	111	24.3
HAZ (height to age) (stunting)	Yes	283	62.1
	No	173	37.9
WHZ (weight to height) (wasting)	Yes	231	50.7
	No	225	49.3
			Percentage (%) or mean (SD)
Underweight			-4.78 ± 2.42
Stunting			-4.49 ± 5.29
Wasted			-2.83 ± 4.49
Child’s chronic medical illness (DM, HIV, and CHF)	Yes	26	5.7
	No	430	94.3

### Prevalence and associated factors of maternal depression

The overall prevalence of maternal depression among mothers of children with undernutrition was 166 (36.4%) with a 95% CI of 32% to 41%.

The following factors were associated with maternal depression at a p-value less than 0.2 according to binary logistic regression: mother’s educational status, marital status, occupational status, history of known chronic medical illness, family history of mental illness, alcohol use, social support, perceived stigma, intimate partner violence (physical and verbal abuse), planned status of the last pregnancy, and on the child: stunting, wasting, being underweight, chronic medical illness, and prior hospitalization.

Multivariate logistic regression analysis indicated that the mother’s educational status, occupational status, poor social support, perceived stigma, and child stunting were significantly associated with maternal depression (p< 0.05) at a 95% confidence interval.

Mothers with a lack of education were 2.9 times more likely to develop maternal depression compared to mothers who had a secondary school education or higher (AOR = 2.872, 95% CI = 1.502–5.492). Mothers who were unemployed were 2.6 times more likely to develop maternal depression than mothers who were employed (AOR = 2.581, 95% CI = 1.497–4.451). Mothers who had poor social support were 2.2 times more likely to develop maternal depression when compared to those who had strong social support (AOR = 2.209, 95% CI = 1.314–3.713). Mothers who had perceived stigma were about 2.24 times more likely to develop maternal depression when compared to those who did not (AOR = 2.243, 95% CI = 1.414–3.560). Mothers of children with stunting were 1.9 times more likely to develop maternal depression compared to mothers of children without stunting (AOR = 1.913, 95% CI = 1.129–3.241) (**Table 6**).

**Table 6:** Bivariate and multivariate logistic analyses of factors associated with maternal depression among mothers of children with undernutrition at a comprehensive specialized hospital in Northwest Ethiopia in 2023 (n = 456).

Variables	Categories	Maternal depression		COR and 95% CI	AOR and 95% CI
		Yes	No		
Mother’s marital status	Married	117	244	1	1
	Separated	17	22	1.611 (.824, 3.150)	.996 (.435, 2.283)
	Divorced/widowed	32	24	2.781 (1.567, 4.933)	1.837 (.925, 3.647)
Mother educational Status	Not educated	52	31	3.302 (1.930, 5.650)	2.872 (1.502, 5.492) ***
	Primary school	50	133	.740 (.475, 1.153)	.824 (.490, 1.385)
	Secondary and above	64	126	1	1
Mother occupation	Employed	41	110	1	1
	Housewife	89	88	1.058 (.620, 1.777)	.928 (.503, 1.712)
	Unemployed/no occupation	36	92	2.713 (1.706, 4.316)	**2.581 (1.497, 4.451)** **
Social support	Poor social support	94	98	2.427 (1.571, 3.751)	2.209 (1.314, 3.713) ***
	Moderate social support	23	68	.597 (.485, 1.524)	.715 (.358, 1.428)
	Good social support	49	124	1	**1**
Perceived stigma	Yes	110	113	3.077 (2.064, 4.586)	2.243 (1.414, 3.560) ***
	No	56	177	1	**1**
Intimate partner violence	Yes	105	99	1.382 (.939, 2.034)	1.380 (.700, 2.720)
	No	190	61	1	1
Kind of abuse	Verbal and physical	35	49	1.429 (.865, 2.360)	1.295 (.715, 2.345)
	Physical	13	19	1.368 (.647, 2.895)	1.204 (.487, 2.979)
	Verbal	28	42	1.333 (.776, 2.290)	1.339 (.680, 2.635)
	No abuse	90	180	1	1
Current substance used	Yes	50	68	1.407 (.917, 2.160)	3.666 (.777, 17.297)
	No	116	222	1	1
Current alcohol use	Yes	30	54	1.267 (.642, 1.745)	1.378 (.748, 2.536)
	No	64	146	1	1
Family history of mental illness	Yes	22	24	1.693 (.917, 3.126)	1.792 (.849, 3.783)
	No	144	266	1	1
Chronic medical history of mothers (DM and HIV)	Yes	16	19	1.521 (.760, 3.046)	1.660 (.690, 3.990)
	No	150	271	1	1
WAZ (underweight)	Yes	140	205	2.233 (1.369, 3.641)	2.837 (.996, 3.387)
	No	26	85	.051	.996
HAZ (stunting)	Yes	119	164	1.945 (1.291, 2.931)	**1.913 (1.129, 3.241)** *
	No	47	126	1	
WHZ (wasting)	Yes	95	136	1.515 (1.032, 2.225)	1.401 (.852, 2.304)
	No	71	154		1
Child chronic medical illness	Yes	15	11	2.520 (1.129, 5.623)	.383 (.142, 1.033)
	No	151	279	1	1
The child had a history of hospitalization	Yes	34	44	1.440 (.878, 2.362)	1.380 (.768, 2.481)
	No	132	246	1	
Last pregnancy planned	Yes	29	74	1.618 (1.002, 2.615)	1.785 (.999, 3.192)
	No	137	216	1	1

*p< 0.05, **p< 0.01, and ***p< 0.001; Hosmer and Lemeshow test = 0.65; and COR, crude odds ratio.

## Discussion

Maternal depression might contribute to undernutrition in children, and consequently, can negatively impact their interpersonal behavior and impair social functioning, leading mothers to be less responsive to their children’s needs. Additionally, depression can reduce a mother’s interest in her child, making it difficult to cope with the demands of motherhood and hindering her involvement in essential caregiving tasks. This lack of engagement ultimately hinders the child’s physical growth.

In this study, the prevalence of maternal depression and its possible associated factors were assessed. The findings indicated that a high number of mothers suffered from maternal depression.

This study showed that 36.4% (95% CI = 32%–41%) of mothers with undernourished children had depression. This is in line with other studies done in Botswana, which found a prevalence of 33.3% (50). However, it was lower than in studies done in Kenya (64.1%), Uganda (42%), and Sudan (41.5%) (10, 51, 52). Variations in the results may be due to differences in the screening tools and cut-off points used across studies. The previous study in Uganda employed the Mini-International Neuropsychiatric Interview (M.I.N.I.), while studies in Kenya and Sudan utilized the PHQ-9, similar to the current study. However, the Kenyan study used a different cut-off point, with a score of ≥5 on the PHQ-9 (51). However, in the current study, the PHQ-9 score was equal to or greater than 10.

In contrast, the current study found a higher prevalence compared to previous studies conducted in North Ethiopia (22.8%) (14), northern Ghana (27.8%) (53), Kenya (27.1%) (54), and Brazil (17.9%) (55).

The possible variation in results may be due to differences in study settings, screening tools, study designs, and sample sizes. For instance, the previous Ethiopian study was community-based, whereas the current study involved a hospital setting focused on severely malnourished children, where maternal depression is likely to be more prevalent. This hospital environment itself could be a contributing factor to emotional distress in mothers. There is another possible explanation: the studies used different screening tools. The previous study employed the Edinburgh Postnatal Depression Scale (EPDS), whereas the present study used the PHQ-9 score (14). In addition, a plausible explanation for the difference could be the difference in sample size between the current study and previous studies.

The current study employed the PHQ-9, a more sensitive screening tool compared to those used in prior studies conducted in northern Ghana, Kenya, and Brazil. These previous studies utilized the Centre for Epidemiological Studies Depression Screening Scale (CES-D) in northern Ghana, the Beck Depression Inventory (BDI) in Kenya, and EPDS in Brazil (53–55).

There are discrepancies in maternal depression rates between our current study and the Brazilian study. The use of the PHQ-9 in our study might have overestimated the prevalence of depression compared to the tool used in Brazil. The other plausible explanation for the discrepancy in the prevalence rate between our current study and the study done in Brazil may lie in the type of research design employed, particularly the cohort used in the Brazilian study (55). In this regard, this study design was performed for a long study period, which may lead participants to develop resilience to adverse conditions of child malnutrition over the longer course of the study period. The current study’s cross-sectional design, with its limited timeframe, offered a minimal opportunity to assess how coping and resilience change over time and influence current depression levels. This one-time survey approach might therefore overestimate the prevalence of depression. Other possible explanations include the socioeconomic status of the participants, maternal education level, and the availability of healthcare facilities in the developing countries. Women in low-income settings, like Ethiopia, often face limited access to quality healthcare and are more susceptible to stress, which can directly or indirectly contribute to the development of maternal depression (56).

Regarding factors associated with maternal depression, mothers with children who did not attend formal education were 2.9 times more likely to experience maternal depression compared to mothers with higher levels of education. This finding supported previous studies conducted in Ethiopia, Saudi Arabia, Iran, and Japan (57–59). Poor readers may experience emotional and behavioral issues that can lead to heightened vulnerability to depression and challenges in managing it (60). Mothers who lack literacy skills suffer from low self-esteem, a lack of confidence, and an inability to make independent decisions, and they are held responsible for every unfavorable event; this leads to negative feelings among mothers. A low education level is a key indicator of poor economic status, being linked to more stressors and a higher risk of developing symptoms of depression (61).

Unemployed mothers were 2.6 times more likely to develop maternal depression than mothers who had been employed. This finding was in line with studies done in Sudan, Kenya, and Saudi Arabia (59, 62, 63). Unemployed mothers often experience psychological distress and anxiety due to concerns about finding work, financial strain, and insufficient income. This lack of employment can further contribute to depression by limiting social interaction and amplifying financial pressures. For mothers in the postpartum period, already a vulnerable time, unemployment can exacerbate existing stress and significantly increase the risk of depression (64).

Mothers with poor social support were 2.2 times more likely to have maternal depression than mothers who had strong social support. These findings are in line with previous studies done in Ethiopia (65–67), Sudan, Pakistan, and Japan (68–70). One possible reason is that mothers who lack social support are more likely to experience stress, loneliness, hopelessness, low self-esteem, and psychological distress (71). Mothers with good social support throughout the postpartum period are better able to handle their responsibilities at home, address challenges and problems, and minimize the risk of major stressors that contribute to depression.

Mothers who had perceived stigma due to being mothers of undernourished children were about 2.24 times more likely to develop maternal depression when compared to those who did not. These findings are in line with previous studies done in Ethiopia (72). Perceived stigma influences self-reported maternal depression or the association between the two. Experienced perceived stigma is a person’s perception of being stigmatized by others because their children are undernourished, which may or may not accurately reflect network members’ behavior and feelings. Internalized perceived stigma is a person’s negative feelings, such as shame and deviance, about their undernourished children (73). The possible justification is the perception of stigma, the belief that people will devalue and discriminate against individuals whose mothers were undernourished. The findings of the current study align well with previous research, which has shown that perceived stigma can negatively impact social support and lead to self-isolation, low self-esteem, and increased vulnerability to stress (74, 75). Perceived stigma predicts higher mental health conditions like depression and social anxiety (76). Mothers of undernourished children are easily subjected to shame, ignorance, feelings of isolation from social situations, and feelings of guilt because their neighbors typically hold them accountable for their children’s undernourishment (72).

Mothers with stunted children were more than two times more likely to have maternal depression than mothers whose children were not stunted. This finding was supported by previous studies done in Kenya, Uganda, Botswana, India, and Pakistan (29, 32, 51, 77, 78). Mothers of stunted children are more vulnerable to stressors related to their child’s health and physical status. This vulnerability stems from feelings of helplessness, sadness, and low self-esteem.

## Limitations

The symptoms of depression were evaluated using maternal recall data from the 2 weeks before the survey; this method may have led to under- or over-reporting of symptoms and could introduce recall bias.

The data were collected through face-to-face interviews, which led to social desirability bias.

We excluded data from mothers whose children had a comorbid developmental disorder.

## Conclusion and recommendation

This study found a higher prevalence of maternal depression among mothers of undernourished children compared to most previous studies. This suggests that maternal depression is a significant burden for mothers caring for undernourished children. The distribution of maternal depression among undernourished children was high among mothers with a lack of education, unemployment, poor social support, perceived stigma, and stunting in children with undernutrition. Therefore, early screening and detection of maternal depression are necessary and can provide educational opportunities, reduce unemployment, strengthen social support, and alleviate perceived stigma among mothers with undernourished children.

Researchers are advised to conduct longitudinal studies to determine the cause-and-effect relationships between maternal depression and children with undernutrition.

## Data availability statement

The original contributions presented in the study are included in the article/supplementary material. Further inquiries can be directed to the corresponding author.

## Ethics statement

The studies involving humans were approved by Institutional review board of University of Gonder. The studies were conducted in accordance with the local legislation and institutional requirements. The participants provided their written informed consent to participate in this study.

## Author contributions

BY: Conceptualization, Data curation, Formal analysis, Funding acquisition, Methodology, Writing – original draft, Writing – review & editing, Investigation, Project administration, Resources, Software, Supervision, Validation, Visualization. BG: Conceptualization, Data curation, Formal analysis, Methodology, Writing – review & editing, Funding acquisition, Investigation, Project administration, Resources, Software, Supervision, Validation, Visualization, Writing – original draft. GoN: Conceptualization, Data curation, Methodology, Supervision, Visualization, Writing – review & editing, Formal analysis, Funding acquisition, Investigation, Project administration, Resources, Software, Validation, Writing – original draft. GiN: Investigation, Methodology, Visualization, Writing – review & editing, Conceptualization, Data curation, Formal analysis, Funding acquisition, Project administration, Resources, Software, Supervision, Validation, Writing – original draft. AM: Data curation, Formal analysis, Visualization, Writing – review & editing, Conceptualization, Funding acquisition, Investigation, Methodology, Project administration, Resources, Software, Supervision, Validation, Writing – original draft. DM: Data curation, Formal analysis, Writing – review & editing, Conceptualization, Funding acquisition, Investigation, Methodology, Project administration, Resources, Software, Supervision, Validation, Visualization, Writing – original draft. MN: Data curation, Formal analysis, Resources, Writing – review & editing, Conceptualization, Funding acquisition, Investigation, Methodology, Project administration, Software, Supervision, Validation, Visualization, Writing – original draft.
